# A case of two shunts in the endovascular treatment of type II Abernethy syndrome

**DOI:** 10.1186/s42155-021-00279-7

**Published:** 2022-01-05

**Authors:** Brenden Bombardier, Adam Alli, Aaron Rohr, Zachary Collins, Kavi Raval

**Affiliations:** University of Kansas Medical Center, 3901 Rainbow Blvd, MS 4032, Kansas City, KS 66160 USA

**Keywords:** Abernethy syndrome, Portosystemic shunt, Portal vein, Shunt embolization, Venous malformations

## Abstract

**Background:**

Abernethy malformation is a rare condition defined by a congenital extrahepatic portosystemic shunt, often leading to absence or hypoplasia of the intrahepatic portal venous system. Although there are no consensus treatment guidelines, interventional techniques now offer minimally invasive treatment options for Abernethy malformations. This case report describes a case of Abernethy Syndrome Type II where the patient had two separate extrahepatic portosystemic shunts treated with endovascular occlusion with two Amplatzer plugs and demonstrates the feasibility of this treatment for this rare condition. This case was in a young adult, adding to the scarce literature of treatment for Abernethy syndrome in the adult population.

**Case presentation:**

We report a case of a 20-year-old female patient with neurocognitive behavioral difficulty, voracious appetite, and chronic encephalopathy secondary to type II Abernethy malformation with not one, but two extrahepatic portosystemic shunts. The patient had failed medical management and was not a liver transplant candidate. Therefore, she presented to us for an endovascular treatment option. The two shunts were treated with endovascular occlusion using Amplatzer vascular plugs. Following embolization, flow into the hypoplastic portal vein improved with near complete occlusion of flow into the portosystemic shunts, thus restoring blood flow into the native portal system. At 3 month follow up, a CT demonstrated complete occlusion of the two portosystemic shunts, and a portal vein diminutive in caliber. The portal vein measured 7 mm in diameter on both pre and post-procedure CT scans. The total volume of the liver was found to be 843 cm3 on pre-procedure CT & 1191 cm3 on post-procedure CT.

**Conclusions:**

This report demonstrates the feasibility of using endovascular embolization to treat Abernethy II malformations. The management strategy of Type II Abernethy Syndrome should be to redirect blood flow into the hypoplastic native portal system, allowing for physiologic hepatic metabolism of splanchnic blood, hypertrophy of the portal system, and growth of the liver from the increased trophic flow.

## Introduction

**Fig. 1:**
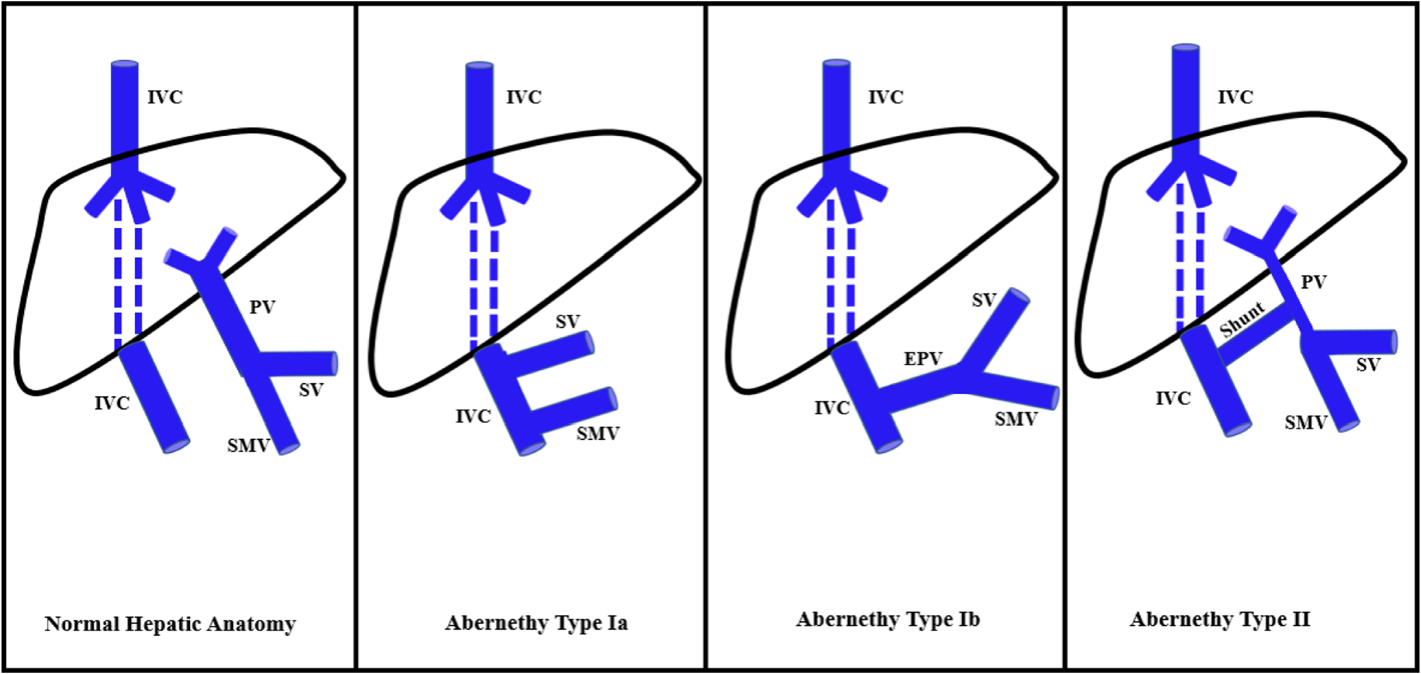
Diagram demonstrating normal hepatic anatomy and hepatic anatomy in Abernethy Syndrome Type Ia, Ib, and II. PV: portal vein, IVC: inferior vena cava, SV: splenic vein, SMV: superior mesenteric vein

Abernethy malformation is a rare condition defined by a congenital extrahepatic portosystemic shunt. Two types of Abernethy syndrome exist: Type I malformations have a congenital absence of the portal vein which leads to complete diversion of portal blood into the systemic circulation. Type I malformations can be further classified into type 1a, where the splenic vein and superior mesenteric vein drain separately into the systemic circulation via the inferior vena cava, and type 1b, where the splenic vein and superior mesenteric vein form a common trunk before draining into the systemic circulation via the inferior vena cava. Type II malformations have a hypoplastic portal vein which leads to at least some of the portal blood flow diverting into the systemic circulation (Fig. 1). (Papamichail et al. 2018; Alonso-Gamarra et al. 2011).

The treatment for Type I Abernethy malformations is liver transplant. (Papamichail et al. 2018; Alonso-Gamarra et al. 2011; Xiang et al. 2019) Although there are no consensus treatment guidelines for Type II Abernethy malformations, they are now commonly treated by endovascular methods. The current literature describes closure of extrahepatic portal shunts using a variety of materials including atrial septal occluder devices, VSD occluder devices, Amplatzer vascular plugs, n-butyl cyanoacrylate, coils, and microcoils. (Papamichail et al. 2018; Venkateshwaran et al. 2014; Rajeswaran et al. 2020; Loureiro et al. 2021; Grimaldi et al. 2012; Suzuki et al. 2013; Yoshimatsu et al. 2006) Rajeswaran et al. retrospectively studied patients with congenital portosystemic shunts treated by either operative ligation, endovascular occlusion, combined surgical & endovascular closure, and observation. They found that at 1 month, serum ammonia levels decreased from 82.5 ± 10.3 μmol/L to 38.4 ± 4.6 μmol/L after shunt closure and no difference was observed in the decrease between patients treated surgically versus endovascularly. Mean occluded to non-occluded portal pressure gradients were significantly lower for endovascular closure (5.3 + − 1.8 mmHg) than for surgical closure (12.3 + − 3.3 mmHg). (Rajeswaran et al. 2020) Symptom regression was reported in most patients following occlusion including improvement in neurologic symptoms and hepatopulmonary syndrome.(Venkateshwaran et al. 2014; Loureiro et al. 2021; Grimaldi et al. 2012; Yoshimatsu et al. 2006; Franchi-Abella et al. 2018) Complications of closure include reported cases of acute portal hypertension and/or thrombosis. However, these resolved with anticoagulation therapy. (Franchi-Abella et al. 2018) This report describes a case of a type II Abernethy malformation with not one, but two extrahepatic portosystemic shunts, treated with endovascular occlusion. This case report was exempt from institutional board approval.

## Case report

**Fig. 2:**
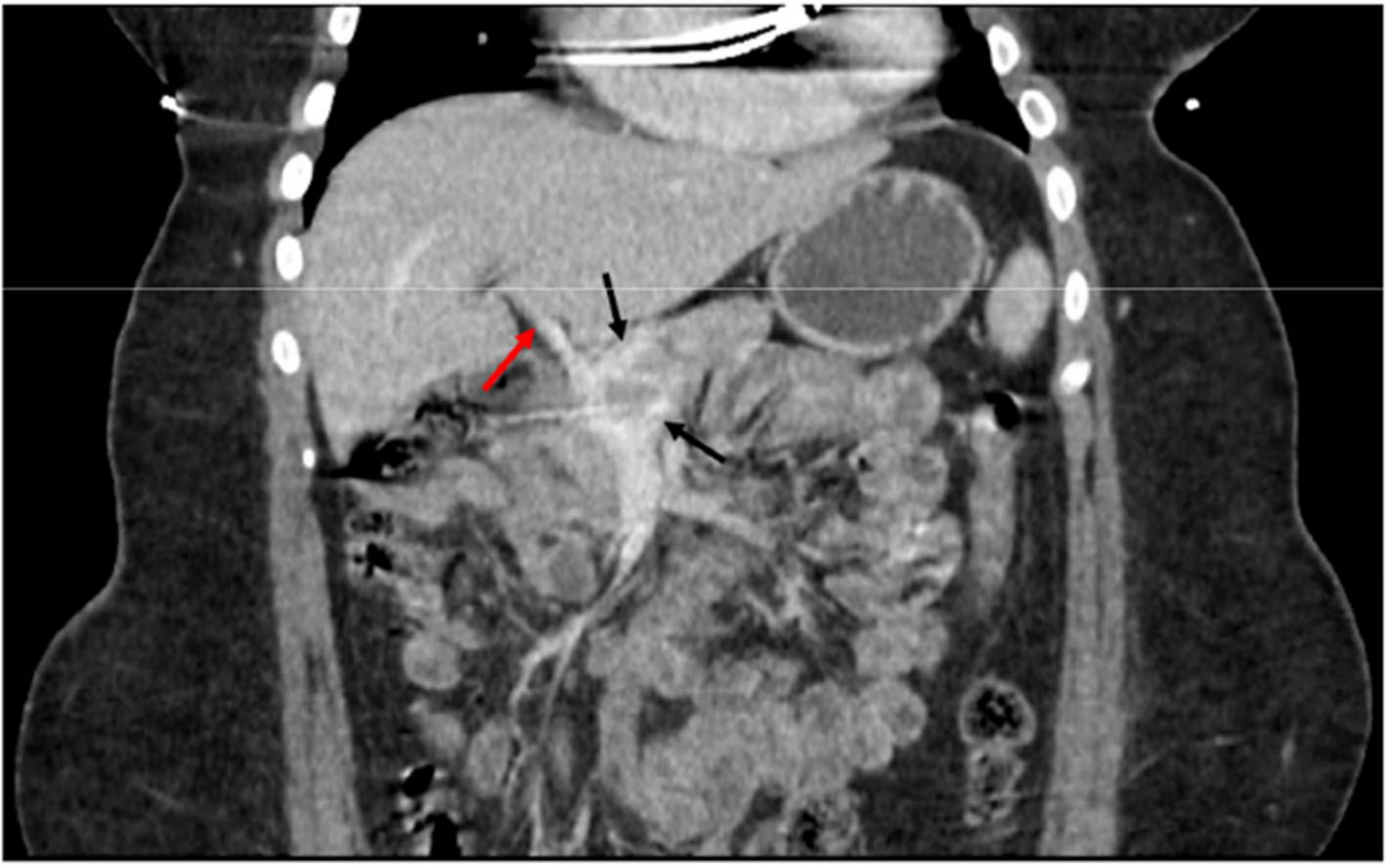
Coronal CT showing two extrahepatic portal shunts (black arrows) and a hypoplastic portal vein (red arrow)

A 20-year-old female patient presented with neurocognitive behavioral difficulty, voracious appetite, and chronic encephalopathy. Despite maximal medical treatment, the patient had persistently elevated serum ammonia levels, with pre-operative levels of 135 and 98 mcmol/L, along with clinical signs and symptoms of encephalopathy. Contrast-enhanced computed tomography (CT) demonstrated a hypoplastic intrahepatic portal venous system. The superior mesenteric vein and splenic vein joined to form a short extrahepatic portal vein, with dominant flow into two parallel shunts into the suprahepatic inferior vena cava (IVC) (Fig. 2). The patient was previously evaluated for candidacy for a liver transplant at a local children’s hospital but was denied eligibility. Thus, the patient and her family agreed to an endovascular procedure to increase blood flow into the intrahepatic portal venous system and decrease blood flow through the portosystemic shunts.

**Fig. 3:**
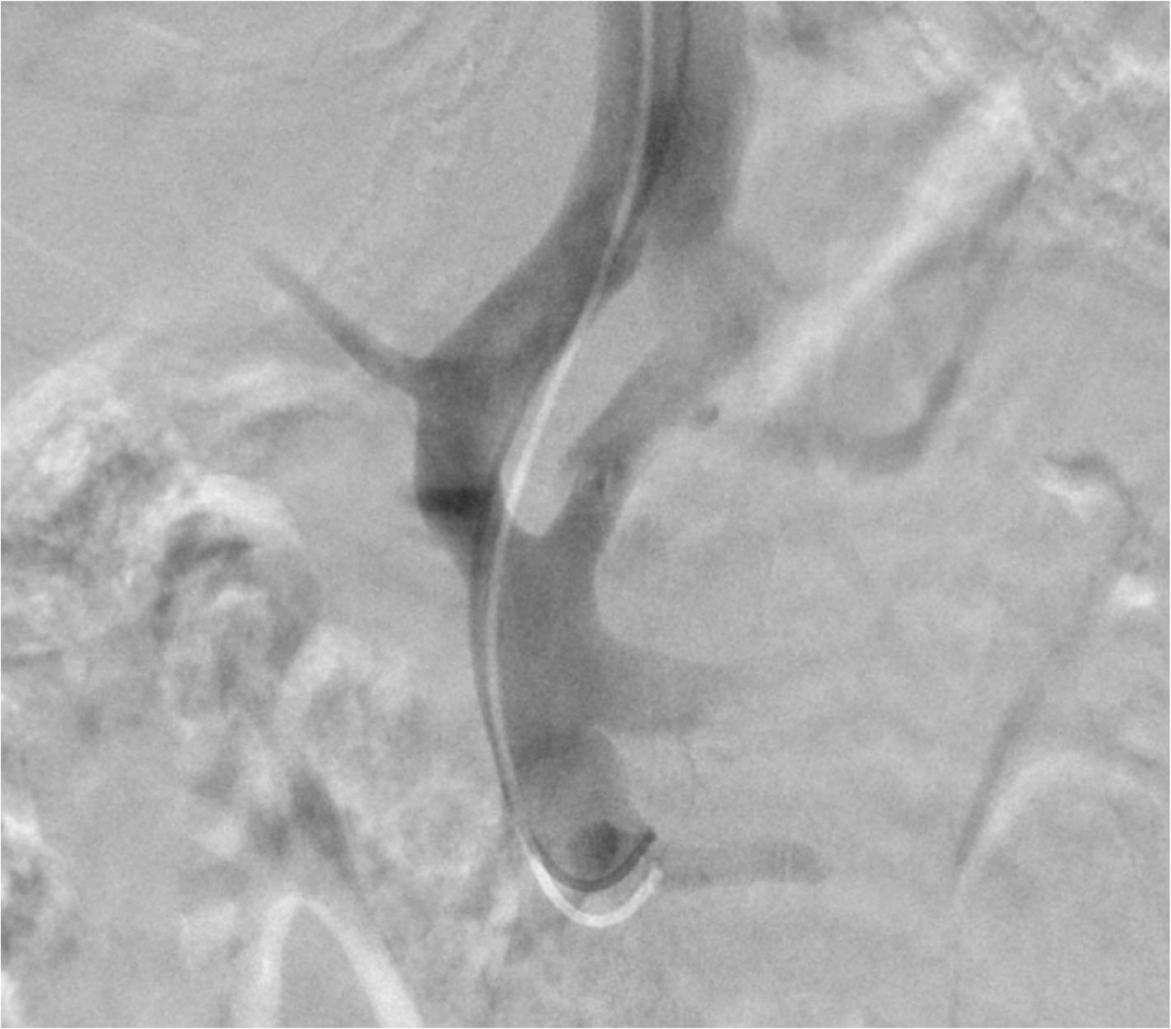
Splenic and superior mesenteric venography showed preferential flow through the shunts into the IVC without flow visualized into the intrahepatic portal vein

**Fig. 4:**
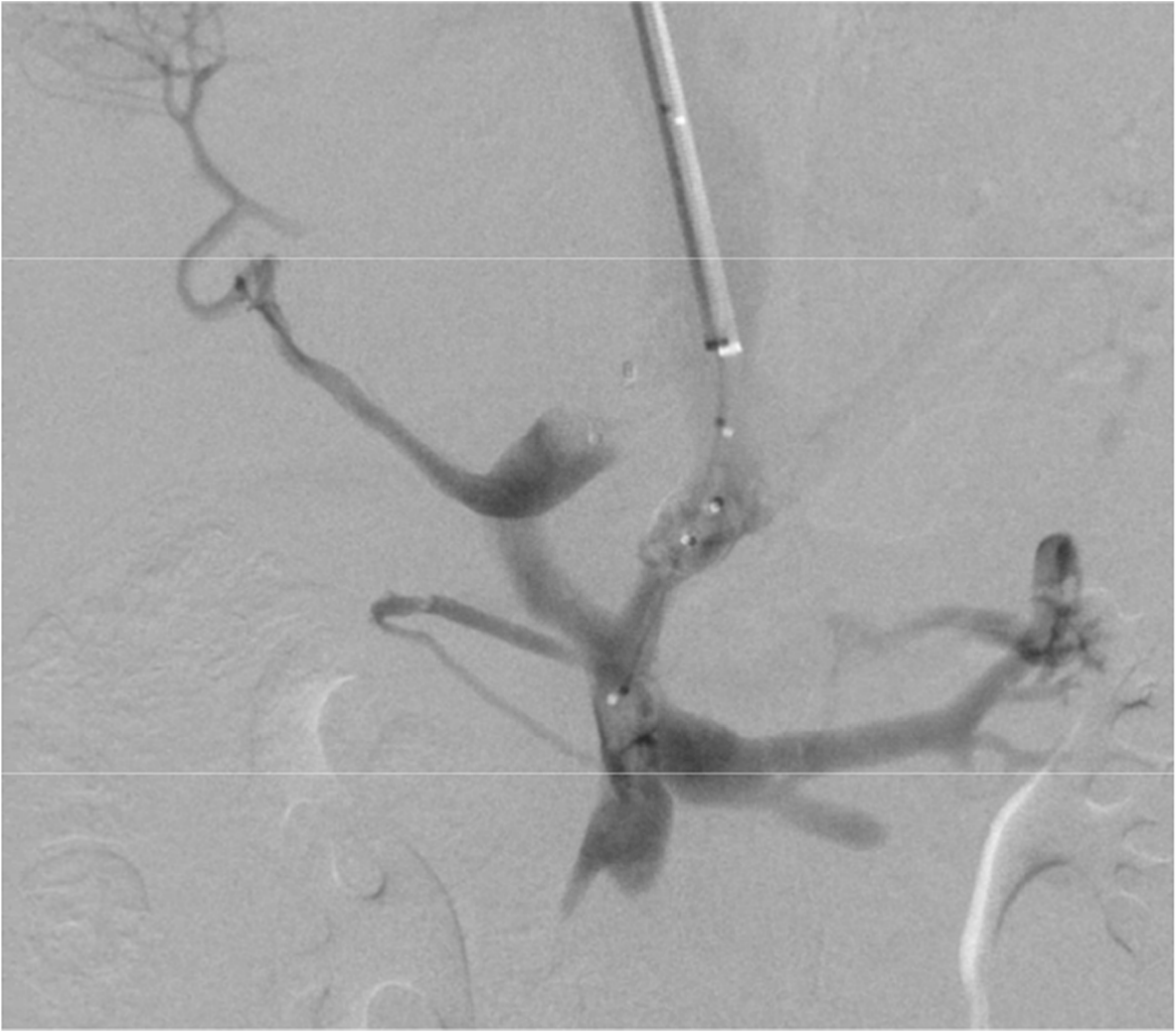
Portal venography following embolization showed increased flow into the intrahepatic portal vein with nearly complete occlusion of the portosystemic shunts

The embolization of the patient’s two extrahepatic portosystemic shunts was performed via a right internal jugular approach. The IVC was selected with an 0.035″ Amplatz wire (Boston Scientific, Marlborough, Massachusetts) with the aid of a 5F JB1 catheter (Angiodynamics, Latham, New York). The catheter was then removed, and a 10F RAABE Flexor sheath (Cook Medical, Bloomington, Indiana) was advanced into the IVC. An inferior vena cavagram was performed, demonstrating wide patency. Next, a JB1 catheter and a .035 angled glidewire (Terumo, Tokyo, Japan) were used to select the right hepatic vein. Digital subtraction angiography (DSA) was performed demonstrating a patent right hepatic vein. The left hepatic vein was then selected, demonstrating a massively dilated vein. Next the portal vein was selected and venography was performed, confirming dominant flow into shunts and a small caliber portal vein. The splenic vein, inferior mesenteric vein, and superior mesenteric vein were all evaluated and were patent with flow into the two portosystemic shunts rather than the diminutive portal vein (Fig. 3). Next, an occlusion balloon (Edwards LifeSciences, Irvine, California) was inflated in the larger shunt to measure pressures prior to plug deployment. The IVC pressure measured 16 mmHg, while the portal venous pressure with occlusion of the shunt measured 18 mmHg prior to intervention. A 16 mm AVPII Amplatzer plug (Abbot, Chicago, Illinois) was deployed in the larger portosystemic shunt. After selection of the second shunt, an occlusion balloon was again inflated, and the portal venous pressure was found to be 18 mmHg. Next a 12 mm Amplatzer plug was deployed. Following embolization, flow into the hypoplastic portal vein improved with near complete occlusion of flow into the portosystemic shunts (Fig. 4). The final portal venous gradient was 2 mmHg.

Enoxaparin was administered at a dose of 40 mg daily for 90 days as prophylaxis against portal venous thrombosis. At 3 month follow up, the ammonia level was 133mcmol/L. Although still elevated, the patient’s family and educators both subjectively noted mild improvements in her behavioral symptoms and described her as being more affectionate and less confrontational. A follow up CT at 3 months demonstrated complete occlusion of the two portosystemic shunts, and a portal vein diminutive in caliber. The portal vein measured 7 mm in diameter on both pre and post-procedure CT scans. The total volume of the liver was found to be 843 cm3 on pre-procedure CT & 1191 cm3 on post-procedure CT (Fig. 1d). The patient had no complications at 3 and 6 months of follow up. At 6 months follow up the patient’s family continued to endorse modest improvement in the patient’s behavioral symptoms. A 1 year follow up abdominal ultrasound showed a normal sized liver measuring 13.1 cm. The main and intrahepatic portal veins were diminutive, though all appeared patent with antegrade flow with a peak velocity of 29 cm/sec in the portal vein. 1 year follow up ammonia level was 97 mcmol/L.

## Discussion/conclusion

This case demonstrated the feasibility and safety of treating two portosystemic shunts with a single staged endovascular closure with Amplatzer plugs. The increase in liver volume post-procedure is consistent with expected increase in trophic flow through the liver following closure of the two extrahepatic shunts. Previous reports describe both single and multi-staged closure, both by open surgery and by endovascular techniques. Previous experience suggests that a single stage approach may be safely performed if the portosystemic gradient remains < 10 mmHg to avoid complications of acute portal hypertension, and the hemodynamics measured in this patient allowed for closure of both shunts without staging. (Rajeswaran et al. 2020)

In conclusion, the management strategy of Type II Abernethy Syndrome should be to redirect blood flow into the hypoplastic native portal system, allowing for physiologic hepatic metabolism of splanchnic blood, hypertrophy of the portal system, and growth of the liver from the increased trophic flow.

## Data Availability

not applicable.

## Abbreviations

CT: Computed tomography
IVC: Inferior vena cava
F: French
DSA: Digital subtraction angiography
